# Supplementary material for: Symptoms of depression among outpatients with suspected COVID-19 in metropolitan Local Government Areas of Kaduna State, Nigeria
Source: PLoS One. 2024 May 16;19(5):e0288567. doi: 10.1371/journal.pone.0288567 (PMC11098515; doi:10.1371/journal.pone.0288567)
Supplement: Attachment — Submitted filename: Response to the Editor and Reviewers Comments.docx [file pone.0288567.s003.docx]

Response to the Editor and Reviewers’ Comments

Data availability: please indicate why data is only available on request. Earlier, it was stated that data is available without restriction. We have revise this and all information pertaining to the study will be uploaded along with the manuscript as supplementary document.Abstract: unclear why “future tense” is used for material and methods; this is a study with results and not a protocol. We have made the necessary revisions.

Methods: Sampling technique - stratified random sampling technique was used. Please define the strata and be explicit about this in relation to the PPS. We made revision and included “The first stratum is the LGA, then the ward (lowest administrative unit), and the type of facility (private or public)”. On PPS we have added “(size of respondents depended on the number of outpatients with symptoms of ARI seen by the facility)”.

Study tools (line 167) – the ARI screening using fever and cough and any of the other symptoms listed, was it a standard screening protocol for ARI? If yes, please provide reference. Yes. We used the standard case definition for ARI. We have provided a reference.

Informed consents: 13yrs are considered minors and consents for them should be different including interviews. Please provide details. We have revised this and added, “Parents or caregivers provided consent on behalf of minors (<18 years), and assent or cooperation obtained from the minors before the interview”.

Results:

Lines 226 – 228: visits to primary versus tertiary healthcare facilities – were equal numbered sampled from these 2 categories of facilities and then screened for ARI? If not, then this statement should be adjusted. We have made the necessary corrections as suggested by the editor.

Recommendation:

Since no causality is inferred, the question readers may have is why trying to identify depression among patients with symptom of ARI? Why not patients with malaria or diabetes or any other illness? The community health officers can be trained to screen all OPD patients for depression. We have this and included, “,,,the Community Health Officers (CHO) and Community Health Extension Workers (CHEW) can receive sufficient training in mental health to enable them to profile all OPD patients for depression and other mental illnesses and refer clients with symptoms of depression to appropriate facilities for better care”.

General observations:

It is difficult to understand the intention of the authors about whether the manuscript is about C19 or other ARI. So much about C19 in introduction and 75% of the discussion is about C19 response, locally and internationally. BTW, C19 is not even part of the title. Though C19 is ARI, but it deserves to be mentioned in the title since it is a special ARI and the authors have devoted almost 75% of the paper to C19. We have made revision and included COVID-19 in the study title, “Symptoms of Depression among Outpatients with Acute Respiratory Infection (COVID-19) in Metropolitan Local Government Areas of Kaduna State, Nigeria”.

Reviewer #1: The article is very useful literary material. It will be clearer if other underlying illnesses such as cardiovascular, disease, asthma and diabetes were used in classifying the subjects, beyond just cases and controls. ARI might not necessarily be the immediate cause of depression. The scope of the study was outpatients who presented with symptoms of ARI (COVID-19), incidentally some of them had other pathologies or diagnosis. We focused more on symptoms of depression, but future studies will explore other diseases.

Reviewer #2: The manuscript has addressed an important research question considering the health indices of the research setting and the socioeconomic status of the study population. The manuscript is also well written in standard academic writing style. However, there are a few concerns the authors need to revise, which are stated below:

ABSTRACT

Background: The background should clearly state the research question, which is the prevalence of depression among patients diagnosed with ARI. We have revised the background and added “We evaluated the prevalence of depression among outpatients diagnosed with ARI”.

MATERIAL AND METHODS

This section should state the measuring instruments used in this study and the study period. A summary of the statistical analysis deployed should also be stated. The research question should be removed from this section and taken to the background. We have revised the study design section of the Methods.

RESULTS

The results is well written. However, the results may be revised to show the proportion of patients that had depression in each class. This way, we should be able to see that people with ARI were more likely to be depressed than those without ARI. It is good that the authors emphasized that causation is not implied by drawing a correlation between ARI and depression. The results have been revised, and we added “and by risk classification, mild (case=15%, control=10%), moderate (case=3%, control=3%), and high (case=1%, control=1%)”.

CONCLUSION

The conclusion is well written. The authors achieved the aim of the study. Thank you.

INTRODUCTION

The introduction should be revised to show the risks and dangers of depression, which justify the study. There is a lot of talk about COVID, ARI, and the epidemiology of COVID. However, the central issue in this study is depression. Why are the authors worried about depression among patients with ARI given that only 5% of people in the general population in Kaduna have this problem? We have revised the introduction and added “Case managers at the isolation centres have observed rising symptoms of depression among COVID-19 patients, and Sensoy et al (2020) had documented a significantly higher average level of depression (24%) among COVID-19 patients [25]”.

MATERIAL and METHODS

Please describe the elements of the questionnaire and if possible, the questionnaire should be included as appendix or supplementary document. We have revised the methods and included “The semi-structured questionnaire has three parts: general information or biodata, questionnaire on symptoms of depression (BDI), and questionnaire on ARI.” We shall upload the questionnaire as a supplementary document. We further described the elements of the questionnaire, “The ARI questionnaire also explored the duration of symptoms, type of health facility visited by patient, clinician seen, diagnosis, recovery and satisfaction with treatment at health facility”.

Indicate if the translators of the questionnaire from English to Hausa were professionals. Also indicate the training the interviewer received. We have also included information on the translation, “The translator is a native Hausa speaker, with over 15 years teaching experience in Hausa and has National Certificate in Education (NCE). The interviewers received intensive 1-day training on the study rationale, questions, design, and tools.”
