# Supplementary material for: Symptoms of depression among outpatients with suspected COVID-19 in metropolitan Local Government Areas of Kaduna State, Nigeria
Source: PLoS One. 2024 May 16;19(5):e0288567. doi: 10.1371/journal.pone.0288567 (PMC11098515; doi:10.1371/journal.pone.0288567)
Supplement: Attachment — Submitted filename: Response to the Editor and Reviewers Comments.docx [file pone.0288567.s004.docx]

**Response to the Editor and Reviewers’ Comments**

Thank you for responding to all comments provided by the Academic Editor and the 2 reviewers. The response provided are acceptable and have addressed most of the issues raised during the review process. However, the edit provided in the title is unacceptable. The revised title reads "Symptoms of Depression among Outpatients with Acute Respiratory Infection (COVID-19) in Metropolitan Local Government Areas of Kaduna State, Nigeria.

Please note that COVID-19 is not synonymous with ARI. The patients have not been confirmed to have COVID-19, they are just presenting with symptoms that may or may not be COVID-19. However, since the period was during COVID-19 pandemic, then they apparently they were COVID-19 suspects. Therefore the tile should be something like "Symptoms of Depression among Outpatients with suspected COVID-19 in Metropolitan Local Government Areas of Kaduna State, Nigeria). Please note that this is just a suggestion to guide the authors and they are not mandated to use this title.

The authors have accepted the editor’s suggestion, and the revised title is "Symptoms of Depression among Outpatients with Suspected COVID-19 in Metropolitan Local Government Areas of Kaduna State, Nigeria.”

The authors are greatly indebted to the editor and the reviewers for their inputs, insights, and suggestions in making the work better. Thank you.
